# Supplementary material for: Active site specificity profiling datasets of matrix metalloproteinases (MMPs) 1, 2, 3, 7, 8, 9, 12, 13 and 14
Source: Data Brief. 2016 Feb 22;7:299–310. doi: 10.1016/j.dib.2016.02.036 (PMC4777984; doi:10.1016/j.dib.2016.02.036)
Supplement: Supplementary file 10 — Supplementary material [file mmc10.zip › WebPICS_hMMP1_T_1%/P1.html]

 

PICS results


|  |  |
| --- | --- |
| **P1\_A**  50 in 277 sites   18.1 %    effects > 10 perc. pnts.  (vice-versa in brackets)  P2prime\_A: 13.0 (25.9)   P2prime\_L: 10.1 (11.4) |  |
| **P1\_C**  6 in 277 sites   2.2 %    effects > 10 perc. pnts.  (vice-versa in brackets)  P2\_C: 29.3 (16.0) |  |
| **P1\_D**  18 in 277 sites   6.5 %    effects > 10 perc. pnts.  (vice-versa in brackets)  P3prime\_H: 13.5 (26.8) |  |
| **P1\_F**  14 in 277 sites   5.1 %    effects > 10 perc. pnts.  (vice-versa in brackets)  P2\_M: 10.7 (14.9) |  |
| **P1\_G**  25 in 277 sites   9.0 %    effects > 10 perc. pnts.  (vice-versa in brackets)  P2\_S: 11.0 (11.0)   P3prime\_V: 12.1 (13.7) |  |
| **P1\_H**  8 in 277 sites   2.9 %    effects > 10 perc. pnts.  (vice-versa in brackets)  P1prime\_V: 29.6 (10.7) |  |
| **P1\_N**  31 in 277 sites   11.2 %    effects > 10 perc. pnts.  (vice-versa in brackets)  P2prime\_M: 11.0 (24.5)   P3prime\_V: 11.5 (16.1) |  |
| **P1\_Q**  16 in 277 sites   5.8 %    effects > 10 perc. pnts.  (vice-versa in brackets)  P2prime\_H: 14.1 (17.3) |  |
